# COST Action ‘ImpARAS’: what have we learnt to improve food allergy risk assessment. A summary of a 4 year networking consortium

**DOI:** 10.1186/s13601-020-00318-x

**Published:** 2020-05-18

**Authors:** Kitty Verhoeckx, Katrine Lindholm Bøgh, Anne Constable, Michelle M. Epstein, Karin Hoffmann Sommergruber, Thomas Holzhauser, Geert Houben, Annette Kuehn, Erwin Roggen, Liam O’Mahony, Ben Remington, René Crevel

**Affiliations:** 1 TNO, Utrechtseweg 48, Zeist, The Netherlands; 2 National Food Institute, Technical University of Denmark, 2800 Kgs. Lyngby, Denmark; 3 Nestlé Research, Lausanne, Switzerland; 4 Department of Dermatology, Medical University of Vienna, Waehringer Guertel 18-20, Room 4P9.02, 1090 Vienna, Austria; 5 Department of Pathophysiology and Allergy Research, Medical University of Vienna, Vienna, Austria; 6 Division of Allergology, Paul-Ehrlich-Institut, Langen, Germany; 7 Department of Infection and Immunity, Luxembourg Institute of Health, Esch-sûr-Alzette, Luxemburg; 8 3Rs Management and Consulting ApS, Asavænget 14, Lyngby, Denmark; 9 Department of Medicine and Microbiology, APC Microbiome Ireland, National University of Ireland, Cork, Ireland; 10 René Crevel Consulting Ltd, Bedford, UK

**Keywords:** Food allergy, Allergy risk assessment, de novo sensitisation, Decision-making criteria

## Abstract

The growing world population and increased pressure on agricultural resources are driving a shortage of dietary protein sources. As a result, industry is developing more sustainable novel food protein sources such as insects, algae and duckweed and using new processing techniques. Consumer exposure to these novel or processed proteins, could cause new food allergies, exacerbating a public health issue which is already directly affecting an estimated 20 million Europeans. Introduction of novel foods should not add to the burden of food allergy and this calls for a reliable, harmonised, evidence-based and validated allergenicity risk assessment strategy. The COST (Cooperation in Science and Technology) Action ImpARAS (Improved Allergenicity Risk Assessment Strategy), a four-year networking project, identified gaps in current allergy risk assessment, and proposed new ideas and plans for improving it. Here, we report on the lessons learned from the ImpARAS network and suggestions for future research. The safe introduction of novel and more sustainable food protein sources, while protecting humans from food allergy, calls for a multidisciplinary approach based on an improved understanding of what determines the relative allergenic potency of proteins, novel testing and assessment methodologies, harmonized decision-making criteria, and a clear ranking approach to express the allergenicity of novel product relative to that of existing known allergenic proteins: (from ‘non’/to weakly and to strongly allergenic proteins).

## Introduction

Forecasts predict a world population of 9 billion by 2050 with a predicted accompanying shortage of proteins for human consumption (WHO/FAO), concurrent with an increasing need to reduce the carbon footprint of agriculture. To address this challenge, strategies are being developed to ensure an adequate, safe, sustainable and nutritious food supply by introducing new protein sources (e.g., insects, seaweed) and expanding and diversifying existing ones. EU regulation 2015/2283 requires that novel foods do not, on the basis of the scientific evidence available, pose a safety risk to human health. To this effect, under the procedure for authorising a novel food and updating the Union list, the European Food Safety Authority (EFSA) should provide an opinion on whether the update is likely to have an effect on human health. In its opinion, EFSA should assess, inter alia, all the characteristics of the novel food that may pose a safety risk to human health and consider possible effects on vulnerable groups in the population. Although for risk assessment of nutritional, microbial and toxicological risks, standardised and well-defined methods are available, methods to assess the risk of food allergy associated with novel proteins are not well-established.

In Europe, an estimated 20 million people have food allergy and the number affected in the community overall (family members, carers, friends, colleagues, etc.) likely exceeds 80 million. This imposes a significant burden of disease on society [1]. The economic impact of food allergy for the food sector and society is substantial, with current estimates for the European health care system alone of over 55 billion EUR annually. Beyond the immediate health costs, individuals with food allergies experience social costs, and the food industry bears economic costs, including allergen management and the financial consequences of food incidents, e.g., recalls due to unintentional presence of food allergens in their products or mislabelling. Social and economic imperatives, therefore, dictate that the introduction of novel proteins should not add materially to the existing large societal burden related to food allergy.

While prevention of future societal costs is an important driver for improved approaches to protein allergenicity risk assessment, the introduction of novel proteins into the diet will be facilitated and costs savings achieved if allergenicity is predicted early in the development process. In particular, this will encourage innovation by lowering barriers for novel foods to enter into the market. Achieving both these aims requires detailed and clear guidance on the assessment of the allergenic potential of novel foods. EU legislators and the EC recognise this need, as demonstrated in Regulation (Preamble Recital 23 in, which states that “Criteria for the assessment of the safety risks arising from novel foods should also be clearly defined and laid down”. However, current guidance relies mainly on a weight-of-evidence allergenicity risk assessment developed for GM-plant foods, which mainly focuses on the impact of a single protein (or at most a few proteins) on individuals with pre-existing allergies and the potential for cross-reactivity. This approach protects individuals with known existing allergies, but it is not applicable for the prediction risks of de novo (new) sensitisation and allergies to novel proteins. For example, Broekman et al. showed that exposure to mealworm can induce de novo sensitisation to larval cuticle proteins, leading to food-allergic responses confirmed in a double-blind placebo-controlled food challenge. The affected individuals were not allergic to any other food [2], in contrast to participants in the same study with allergies to crustacea, which were well-predicted and confirmed to be food allergic to mealworm proteins in addition to that to Crustacea. The culprit proteins were not identified as allergenic using the homology testing strategy as proposed by CODEX Alimentarius Commission guidelines and endorsed by EFSA. Another study identified newly introduced epitopes after deamidation of gluten, which could be responsible for the severe allergic reactions after consumption of deamidated gluten in people tolerant to unmodified wheat products [3]. In conclusion, these examples highlight the importance of assessing the de novo sensitisation potential of novel and processed proteins and addressing it in the risk assessment of novel foods. Such an assessment would complement the allergenicity assessment with regard to potential allergenic cross-reactivity and permit a complete prediction of allergenicity. But, as indicated before, methods for this are largely lacking and this knowledge gap was therefore, one of the two main drivers of the COST (Cooperation in Science and Technology) Action ImpARAS.

## ImpARAS

ImpARAS is the acronym for a COST Action entitled “Improved Allergenicity Risk Assessment Strategy” (FA1402, www.imparas.eu). COST Actions are bottom-up, pan-European research networks funded by the various research and innovation framework programmes, such as Horizon 2020. Funding was not for research itself but for networking, training and dissemination activities.

Representatives from different sectors including industry, academia, risk assessors, regulators and clinicians from 30 European countries took part in many ImpARAS activities over the past 4-years. Besides 4 annual conferences held in Belgrade, Elsinore, Naples and Warsaw, ImpARAS organised 3 training schools (allergenicity risk assessment, proteomics in allergenicity assessment and animal models in allergenicity assessment), 7 Working Group meetings (Barcelona, Madrid, Milan, Nantes, Porto, Utrecht, and Vienna), 3 stakeholder meetings in Brussels with members from the European Commission, patient organisations, food industry, food safety authorities, risk assessors and lawyers. The network published > 25 peer-reviewed papers (see Table 5) and facilitated the exchange of 37 early stage researchers between different European institutes.

All information (abstracts and presentations) can be found on the ImpARAS website (www.imparas.eu).

The aim of ImpARAS was to build an interdisciplinary network of researchers to better understand the mechanisms of allergy and develop new ways to assess the allergenicity of novel proteins (see Table 1). The basic question: ‘what makes a food protein weakly or strongly allergenic?” sums up the purpose of ImpARAS, which focused on identifying and characterising both the intrinsic difference between an innocuous food protein, unlikely to generate an allergic response, and a potential food allergen as well as any factors which may modulate this difference to better predict allergenic risks associated with novel or modified food proteins. ImpARAS recognised that de novo development of an IgE-mediated allergy resulted from a combination of different elements and factors (proteins and product characteristics, the host individual’s characteristics, and environmental factors), however the focus was mostly on the protein itself. Beyond the development of new insights and methods, ImpARAS recognised the crucial importance of relating them to risk assessment approaches, such that they would be fit to address the risk management questions raised by the introduction of novel proteins. The ImpARAS network consisted of 4 Working Groups (WG) that worked closely together and extensively exchanged knowledge during the course of the Action. The tasks assigned to the 4 working groups are listed in Table 1.

**Table 1:** Objectives of ImpARAS and tasks assigned to the 4 ImpARAS working groups

Working group	Tasks and objectives
ImpARAS	Building a European network of leading institutes undertaking basic research on food safety, food allergy and allergy risk assessment to strengthen the international competitiveness of the European scientific community on this topic Generating ideas on an improved risk assessment strategy to determine the allergenic potency of (novel) and/or processed proteins Generating ideas for the development of new more predictive tools/methods for allergenicity Disseminate the knowledge acquired to the European food industry leading to the development of novel safe food products and to the European food safety authorities to improve their allergy risk assessment strategies
WG1	Review analytical methods used in allergenicity assessment and identify methods relevant for an improved allergenicity assessment Refine appropriate protocols for purification of allergens Identify physicochemical properties of proteins that may affect sensitisation Investigate which proteins (allergenic and non-allergenic) can be used in in vitro and in vivo allergenicity assessment studies
WG2	List in vitro methods (or combinations thereof) that can be used to predict the sensitising capacity of a protein Investigate the possibilities of harmonising and validating in vitro models
WG3	Identify which (combination of) species, can be used to predict protein allergenicity in humans Identify reliable end-point parameters that can be used to predict for sensitisation Investigate the possibilities of harmonising and validating in vivo models
WG4	Identify the gaps in the current allergenicity risk assessment strategy Implementation of WG1-3 findings in an improved risk assessment strategy Involvement of regulatory authorities in the new concept and dissemination among food companies

### WG1: physicochemical properties of proteins impacting allergenicity

Proteins foreign to an individual, such as those found in food, normally provoke a response when encountered by the immune system. However, even in the case of food allergens, only in a small proportion of predisposed individuals does this response progress into a pathological, allergic response. It is currently unknown why certain proteins are allergens, whereas others are not and what characteristics drive a protein to provoke an allergic immune response. WG1 focussed on physicochemical properties of proteins that may influence sensitisation (i.e., the production of allergen-specific IgE antibodies) and are a potential parameter for predicting the allergenic potency of a protein.

In the review paper of WG1 [4], the group re-evaluated the existing procedures for allergenic risk assessment of GMOs for their application to novel foods, identified the gaps in allergenic risk assessment of novel foods and made recommendations for further research (see Table 2). The review highlighted that comparative material and tests specific for novel and potentially allergenic structures are lacking. The review also summarised and critically evaluated the currently available analytical methods for purification of allergens from their natural sources and recombinant production of allergens, including the assessment of primary, secondary and tertiary structure of allergens. WG1 also provided a critical evaluation of strengths and limitations of each of these methods, highlighting the gaps of the current allergenic risk assessment. In parallel, the immunoassays testing allergenic activity of proteins were summarised including two tests addressing the capacity to bind IgE-antibodies and assays assessing the reactivity as mediated by IgE on the surface of effector cells.

**Table 2:** Gaps identified in the current risk assessment and recommendations for further research. (Reproduced with permission from the authors [4] Copyright 2017, Published by Wiley-VCH)

Methods and tools	Features and limitations	Recommendations for further research
Allergen databases	Different databases provide different levels of information; Some of them are not regularly updated/curated and therefore relevant information is missing or available information outdated; Inclusion criteria for allergenic proteins vary for individual databases	Linking of existing (allergen) databases; Harmonisation of inclusion criteria for allergens; Experimental studies in B- and T cell epitopes and implications on cross- reactivity; Improving predictive algorithms for sensitising potential of proteins linked with and without clinical relevance;
Analytical methods	Highly sensitive and advanced methods available for protein characterisation; Sample preparation especially for complex food extracts is sometimes difficult (lack of harmonised protocols);	Harmonisation of method protocols; Improvements in sample preparation; Generation of scientific evidence of certain structural determinants (glycosylation, aggregation etc.) linked with increased allergenicity, which is currently lacking;
IgE binding assays	Well standardised reference assays including reference proteins are missing. In case of novel proteins, no reference material is available; If sIgE is not available, animal-derived antibodies can be used;	Identification and generation of suitable reference proteins;
Digestion assays	Different protocols for protein digestion are available; However, harmonised protocols are needed; Lack of guidance how to interpret data, and lack of reference material; Evidence of linking protein stability and de novo sensitisation is missing;	Development of reference materials and harmonised protocols; Performance of harmonised digestion assays in ring trials with reference materials; Animal studies on comparative digestion and de novo sensitisation;
Food processing techniques	Knowledge on food processing and its impact on allergenicity is incomplete on a qualitative and quantitative level. Limited knowledge about the most effective methods (combinations), including novel processing techniques;	More data on processed food proteins and their allergenicity required; To identify the most important (combination of) processing techniques with an impact on allergenicity;
Food matrix	Analytical methods are established—but limited data are available showing a link of food matrix components to allergenicity; Limited knowledge available about food components and their interaction with allergens;	Studies required on food matrix composition and interaction with individual food proteins in model systems; Identification of relevant immunomodulating food matrix components;
Biological assays	Cellular and animal models are established but reliable assays for detection of de novo sensitisation are lacking	Method development to assess protein ligand binding and impact on innate and adaptive immune responses; Identification of biomarkers for de novo sensitisation

WG1 discussed the use of protein pairs (a combination of an allergenic and a homologous non/weak allergenic protein) in order to develop potential comparators/calibrators for future allergenicity assessment. Using well-characterised patient cohorts, the group investigated the applicability of the tropomyosin protein pair from shrimp (allergenic) and from chicken muscle (non/weak allergenic) (Kleuber, see Table 5) and the protein pair of beta-parvalbumins (allergenic) and alpha-parvalbumins (non/weakly allergenic), respectively (Kalic, see Table 5). Both pairs were used in immunoassays such as BAT and IgE binding tests (ELISA, immunoblot) and proved suitable (at the level of cellular testing) as a potential novel approach in allergenicity testing of potentially cross-reacting novel foods. Notably, this should be done in titrated assays targeting the comparison of allergenic potency. The concept of “pair of allergens” was further developed by expanding it to homologous series of proteins with different allergenic potency, to work towards a refined toolbox of parameters relevant for allergenicity assessment, and applying methods that are calibrated with meaningful benchmark comparators of high and low allergenicity.

Furthermore, WG1 collated and analysed certain physicochemical parameters of relevant plant and animal derived allergenic proteins. Several parameters (e.g., glycation, glycosylation, lipid binding, phosphorylation, aggregation) were investigated for the most representative plant families of allergens, such as legumins, vicilins, 2S albumins, nsLTPs, PR-10 proteins and profilins of the plant allergens, or e.g., caseins, parvalbumins and tropomyosins in the case of animal allergens. This extensive study provided a good overview on the different physicochemical parameters and their relevance for different protein families of plant and animal food allergens. Certain parameters, like heat stability, resistance to proteolytic activity and structural stability are considered important for protein allergenicity, but clinical data directly linked to these parameters are lacking (Costa in prep, see Table 5). These WG2 reviews showed that no single distinct molecular parameter (or pattern) found within one protein family is exclusively responsible for the allergenic potential at the site of elicitation. However, continued detailed characterisation of allergens may further elucidate molecular patterns, such as those presenting with intrinsic adjuvanticity, and that further stimulate the immune system towards an increased efficiency in sensitization against the allergenic protein.

### WG2: in vitro methods to predict sensitisation

Many cells of the immune system are involved in allergic sensitisation (e.g., epithelial cells, dendritic cells (DCs), T- and B-lymphocytes) and elicitation of symptoms (basophils and mast cells). Basophils and mast cells are often used to determine functional IgE binding, for instance, to test the allergenicity of an allergen after thermal processing or enzymatic hydrolysis (hydrolysed milk formula). They are also used to identify cross-reactivity of a novel or GMO protein with known allergens. However, these cells are not suitable for the identification of de novo sensitisation. Epithelial cells, DCs and T cells are often used to study immunological reactions and mechanisms, but are hardly used in allergenicity assessment. To date, there are no in vitro methods available for predicting sensitisation.

**Fig. 1:**
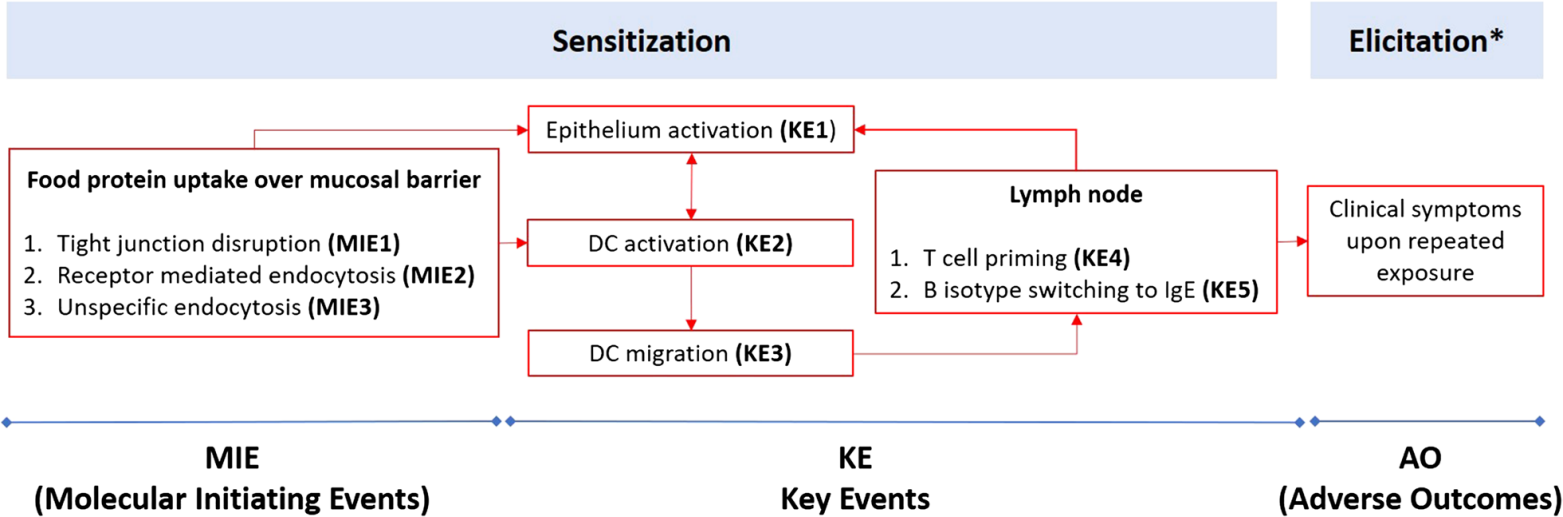
Adverse Outcome Pathway for food sensitisation

WG2 reviewed existing and emerging knowledge concerning protein uptake and bioavailability, the activation of the innate and adaptive immune mechanisms and processes (e.g., DCs, innate lymphoid cells type 2 (ILC2), T cells, iNKT cells, antibody class switch), and the importance of the route of allergen exposure. The available information was curated for relevance and quality and the retained molecular and cellular mechanisms were assessed for plausibility and structured according to the Adverse Outcome Pathway (AOP) concept (van Bilsen, see Table 5) (Fig. 1). The proposed AOP was accepted as a non-OECD work plan project by OECD (http://www.saaop.org/).

Using the key events (KE) that emerged from the AOP for guidance, the group evaluated different in vitro tools for their ability to provide information on the biological effects of allergenic protein that is useful for hazard identification and eventually contributes to risk assessment and human safety. The identified models were grouped according to the molecular initiation event (MIE) or key event of the AOP they addressed, and discussed for their potential relevance in determining the sensitising (incl. sensitising route) properties of new foods and food proteins (Table 3) (Lozano, see Table 5). It was deemed that a first evaluation project should focus on methods addressing MIE 1-3 and KE1 using the most frequently used epithelial cell models, preferably human epithelial cells.

**Table 3:** Methods addressing several MIE’s and KE’s shown in Fig. 1

Event	In vitro method	Read-outs
MIE 1, 2, 3 & KE1	M cells	Allergen quantification (SDS-PAGE, Western blot and microscopy) Integrity of ZO-1 (microscopy).
	T84	Monolayer integrity (TEER) Cytokine production (ELISA)
	HCT-8	Monolayer integrity (TEER) Cytokine production (ELISA)
	Caco-2	Monolayer integrity (TEER and Lucifer Yellow) Allergen quantification (ELISA, SDS-Page, Western blot and LC–MS,) Integrity of A20 (Western blot and RT-qPCR blot and microscopy) Allergen transport (RBL activation test) Gene expression (RT-qPCR)
	HT-29	Monolayer integrity (TEER). Allergen quantification (ELISA) Integrity of A20 (Western blot and RT-qPCR
KE 2 & 3	Mouse BM-DCs	Allergen uptake (flow cytometry) Migration assay (flow cytometry) Cytokine production (ELISA) DCs maturation (flow cytometry)
	THP-1-derived DCs	Allergen uptake (flow cytometry) Gene expression (RT-qPCR) Cytokine production (ELISA)
	Human Mo-DCs	Expression of DC markers (flow cytometry)
KE 4	Human T cell clones	T cell proliferation ([^3^H]-thymidine) Cytokine production (ELISA) T cell activation (flow cytometry)
	Human peripheral blood mononuclear cells (PBMCs)	T cell proliferation (CFSE or [^3^H]-thymidine) Cytokine production (flow cytometry) T cell activation (flow cytometry) Gene expression (RT-qPCR) Expression of T cell markers (flow cytometry)
	Mouse MLN-isolated T cells	Cytokine production (ELISA)
	Mouse LP-isolated mononuclear cells	T cell proliferation (CFSE) Cytokine production (flow cytometry)
KE 2, 3 & 4	Co-culture: BM-DCs/primed T cells	Allergen uptake (flow cytometry) T cell cytokine production (ELISA) DCs maturation (flow cytometry)

### WG3: in vivo methods to predict sensitisation

To fully evaluate the potential sensitising capacity of novel foods, the development of suitable animal models that provide a more holistic assessment of the allergenic potential of novel proteins is currently still required and recommended. Although a variety of animal models have been proposed, none have been validated, or widely accepted. The choice of animal species, experimental design as well as the selection of appropriate endpoints parameters may lead to contradictory results, thus resulting in an enormous impact on performance and predictive accuracy of animal models.

**Fig. 2:**
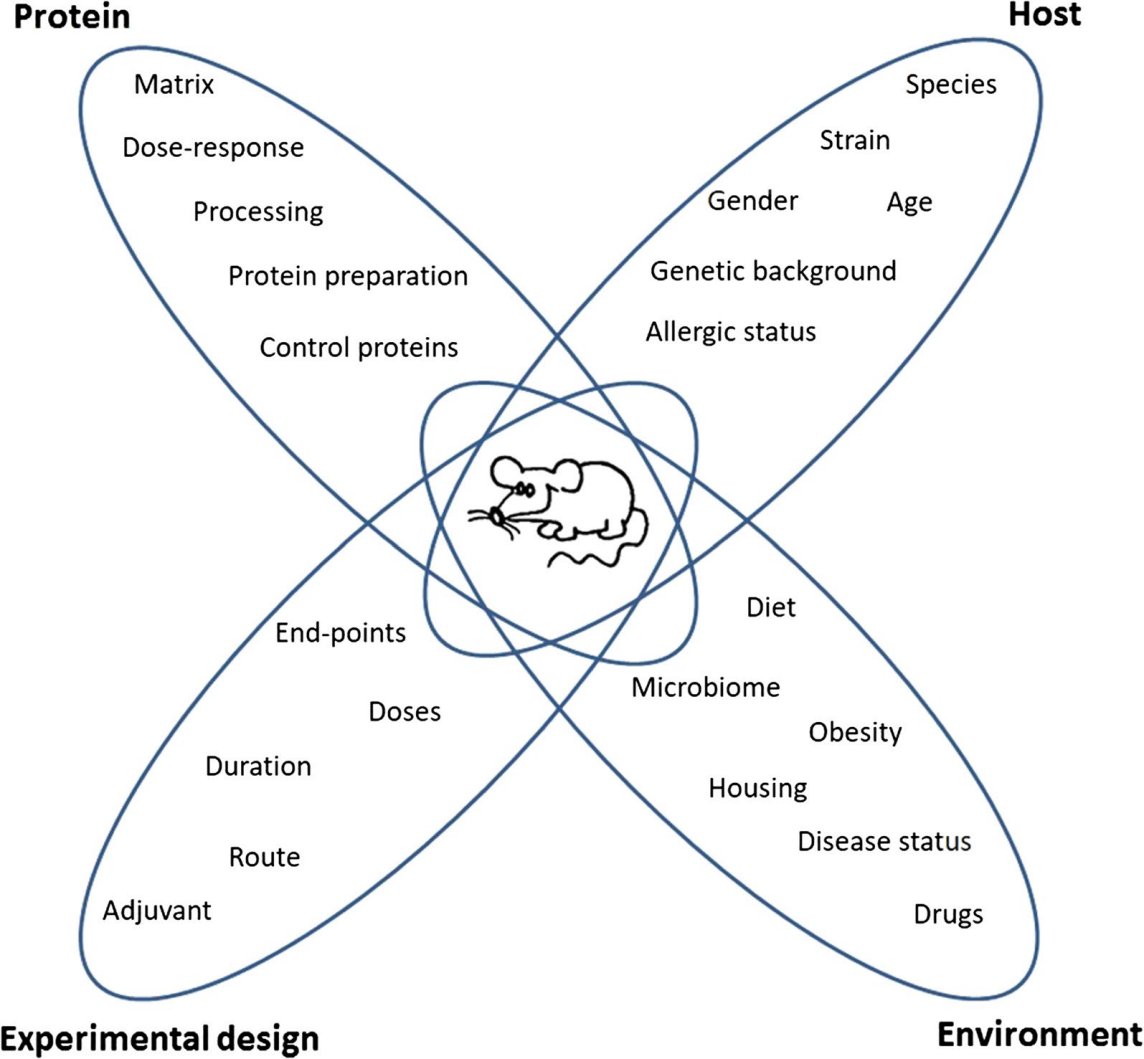
Parameters to consider when designing animal models for assessing the sensitising capacity of food allergens. These parameters are related to either the protein, host, experimental design or the environment Modified from Bøgh et al. Clin Transl Allergy 2016 [5]

WG3 extensively reviewed and examined important aspects in the design, conduct and interpretation of animal models for assessment of the allergenic potential of novel food proteins, which is summarised in Fig. 2, [5]. In the review, the group stated that in a good model, multiple doses of a novel protein should be assessed and the novel protein should preferably be tested in a relevant food matrix and not as an isolated protein to control for matrix effects. Furthermore, measurement of IgE induction alone will not be sufficient to determine allergenicity, the use of additional endpoint parameters such as mucosal responses (e.g., cell infiltration) or serum inflammatory mediators (e.g., mast cell products) are needed and assessment of in vivo responses to protein challenge (such as change in temperature or ear swelling) is recommended and highly advisable. The group suggests performing a ring trial across multiple laboratories, to address animal model stability and transferability and to identify the factors (e.g., animal house microbiome) that impact replication of studies between different laboratories. WG3 wrote a proposal for a ring trial based on the need to improve the reliability of the models and the ability to compare them. The group suggested to base the predictive animal model on a mouse model presented by Smit et al. because of the need to establish a simple and cost-effective model, that would not require the use of a large amount of protein [6]. To evaluate and rank the sensitising capacity of tested foods/proteins, a series of evaluation parameters should be used to improve the reliability of the model.

Another important aspect to consider when developing a harmonised and validated animal model, is the use of positive and negative reference control proteins (cf WG1). In addition, the design of the animal model and execution of the experiment as well as the measurement of the quantitative and qualitative evaluation endpoint(s) are important for validation, harmonisation and reproducibility of animal models.

A special focus was on identifying the optimal ex vivo and in vivo disease endpoint parameters and their relevance when assessing potential allergenicity of novel foods in experimental animal models. The main conclusions of WG3 were published in a review paper (Castan, see Table 5) which emphasised that the choice of endpoint parameters, the quantity of different endpoints evaluated, and the exact technology used are essential for the outcome and predictive reliability of the model. A key point is that each endpoint has its strengths and limitations. The paper reviewed the use of temperature, level of Ig’s, phenotyping of the cell infiltrate and cytokine production, and stated that these endpoints provide information about the allergic reaction and the degree of sensitising capacity of the allergen. However, the endpoints do not always provide a complete understanding of the immune mechanisms underlying the allergic reaction and thus, there is a strong need to elucidate these mechanisms in order to predict the clinical outcomes of sensitisation to novel food proteins. For example, the current experimental animal models are not able to predict the magnitude of the allergic response to a particular allergen. To improve allergenicity risk assessment with the use of experimental animal models, it is necessary to discover which cells and molecules are essential for the sensitisation and elicitation phases of food allergy.

### WG4: risk assessment and dissemination

The first task of WG4 was to review current guidance and the existing strategies to assess the potential allergenicity of new proteins introduced into the diet, and to assess the adequacy of the strategies to assess risks associated with de novo sensitisation of IgE-mediated allergy (Remington, see Table 5). It was concluded that the existing tools and tests are capable of adequately assessing potential cross-reactivity and research is available regarding predicting cross-reactivity for complex novel foods [7]. Current approaches that have been applied for assessing safety through a weight-of-evidence approach to approve GM crop commercialisation appear to be well suited to protecting consumers. However, the level of exposure to novel proteins in these products is low in comparison to that expected for proteins used for nutritional reasons. Novel foods intended as protein sources may thus pose different challenges, in that complex mixes of proteins are expected, and exposure scenarios will be very different. As summarised by (Remington, see Table 5), the other WGs and other ImpARAS publications (Table 1), there are few methodologies available that are applicable for a strategy to identify and characterise, with reasonable certainty, the risks arising from de novo sensitisation and there is no single test available (or expected in the near future) for predicting or characterising the de novo sensitisation potencies of new proteins, let alone complex mixtures.

In order to facilitate a coherent allergenicity risk assessment strategy, a clear outline of preferred decision-making criteria is needed from the risk management sector. The decision-making criteria would help guide researchers during method development and ensure the applicability of newly developed methods for critical risk assessment questions. Therefore, a second WG4 activity was instigated to define possible risk management targets for the assessment of IgE-mediated allergenicity of new or modified food proteins and their implications on future methods development. Briefly, for example, if a hazard identification based criterion in the sensitisation phase would be the risk management decision point (i.e. is the new protein a sensitiser or not?), in principle, this criterion would provide the most extensive consumer protection (but likely over-protective in practice). It implies that no protein would be accepted that has any potential for sensitisation, and means that every new food would have to be safer than any existing food. It is questionable whether non-sensitising proteins exist or if methods allowing verification of this criterion could ever be achieved. To further illustrate the concept, case studies of novel protein food products (chia, rapeseed protein isolate and ice structuring protein) and the respective EFSA risk assessments were analysed. From these case studies, even for a protein without any history of sensitisation or elicitation of allergy symptoms, a residual potential for causing sensitisation and allergy was not excluded. This seems reasonable from a methodological point of view (the absence of something cannot be proven), but questionable when considering the existence of fully non-sensitising or non-allergenic (food) proteins. It is, therefore, unlikely that a qualitative binary hazard-based criterion such as “non-sensitising/sensitising” can effectively be used. Alternatively, quantitative hazard-based criteria (“weakly sensitising/strongly sensitising” or “low eliciting doses/high eliciting doses”) may be feasible to implement, but realisation would depend on the consensus establishment of a cut-off value to discriminate between weak/strong and high/low sensitisation or elicitation. This conceptual approach and options of possible decision-making criteria were presented in a stakeholder meeting in Brussels on February 2018 and were recently published (Houben, see Table 5). The published criteria, however, are preferably not used individually, but in combination with the expected level of exposure. In the case studies for novel food applications, it was argued that sensitisation, allergy or the elicitation of allergic symptoms would not be expected at the anticipated levels of exposure or after implementation of appropriate risk management measures. In all cases, the proven or potential existence of an evident hazard was accepted and a risk-based decision was made, aiming to reduce the potential risk to a zero- or minimal level. Admittedly, there was no explicit acceptance of a certain level of risk by the risk assessors or risk managers (a definite type or frequency of sensitisation, allergy development or elicitation of allergic symptoms). Because of the importance of exposure within a risk assessment, a third WG4 activity was initiated to investigate the feasibility of future research to enable and allow the establishment of generic exposure thresholds for allergic sensitisation. For instance, a generic threshold below which allergic sensitisation to a protein was highly unlikely to occur could function in a manner similar to the Threshold of Toxicological Concern (TTC). This would permit the use of proteins at very low levels of intake to proceed with much more limited, if any allergenicity testing, thereby facilitating their introduction without detriment to public health. The feasibility of approaches to establish these threshold levels is being investigated by WG4. As a first step, the feasibility of estimating dietary exposure to single proteins, i.e. specific allergens known to sensitise via the oral route and proteins not known to sensitize via the oral route at existing levels of exposure were examined. The ultimate aim would be to establish an intake level of any protein below which no sensitisation is known to occur, and could be applicable to enzymes, residual proteins in novel food extracts, or proteins in GM products, reducing any need for further allergenicity assessments. A peer-review hypothesis paper is in preparation on this topic.

### Main conclusions of ImpARAS and future perspective

The current allergenicity risk assessment strategy for novel foods is based on the GMO guidance, but is inadequate to characterise the allergenicity of novel food protein sources. The ability of a novel protein to provoke reactions due to cross-reactivity in individuals with pre-existing allergy can be identified and quantified. However, current approaches cannot predict or quantify the potential for de novo sensitisation or allergy induction to a protein. Additionally, there is a lack of clarity about risk assessment and management criteria, which has held back the development of methodologies crucial to risk assessment questions and should urgently be addressed before targeting method development.

Human allergic responses are complex and there is a need for a comprehensive, systematic testing and assessment strategy to identify, characterise and rank the risks associated with allergic reactions due to de novo sensitisation. Future research may provide more insights into why some proteins are more allergenic than others and may increase the possibilities for quantitative risk assessment. The introduction of new protein sources that improve the sustainability of our food protein supply will lead to consumption at high, nutritionally relevant intake levels, and risk assessment approaches aiming at zero-risk will not work under those circumstances. Certain levels of risk will have to be accepted. Projecting the potential allergenic risks of new protein products against a benchmark of risks of known allergenicity of existing foods will help to set relative risks in the context of existing foods and support regulators in decision-making. With advances in scientific knowledge, it will be possible to improve the methodologies used in allergenicity risk assessment, e.g., new possibilities resulting from the development of refined bioinformatics tools, and relevant in vitro tests. It will be vital to identify approaches, methods and technologies on which future research efforts should be focused, considering their current performance, and the scope of their evolution into predictive risk assessment approaches for risk assessment and management decision criteria set by risk managers. A better understanding of AOPs could guide the development of better in vitro and in vivo allergenicity testing methods. Therefore, it is important to regularly review and update regulations and guidelines to acknowledge new knowledge and methodologies.

The main conclusions and recommendations of the ImpARAS network are further highlighted in Table 4.

**Table 4:** Main conclusions and recommendations where future research should focus

1	The ImpARAS STSM programme was very fruitful in achieving its objectives of fostering collaborations between individuals and institutions, many of which endure beyond the Action. A network of expertise covering core aspects of immunology, food allergy, protein chemistry, bioinformatics, proteomics and risk modelling is needed to enable and support integrated risk assessment models and strategies well beyond the current state of the art *How: Through members of ImpARAS with support of COST organisation and experience of earlier COST Actions* (e.g*., INFOGEST*)
2	A clear outline of preferred decision-making criteria is needed from the risk management sector to help guide researchers during method development and ensure the applicability of newly developed methods to the risk management questions at hand *How: Stakeholder working group and workshop*
3	There is a need for agreement/consensus on a comprehensive, systematic testing and assessment strategy to identify and characterise the risk of de novo sensitisation and allergic reactions to novel food proteins, which incorporates relevant aspects of exposure, intrinsic protein properties and matrix/processing effects *How: Workshop developed through ImpARAS consortium*
4	In vitro methods should focus on the different events of the AOP for food allergy sensitisation and initially, especially MIE 1-3 (food protein uptake over mucosal barrier) and KE1 (epithelium activation) using human epithelial cell models *How: European*-*funded research project*
5	In vitro and in vivo methods including clear endpoint(s) need to be harmonised and validated for instance in ring trials using specified reference proteins/extracts *How: European*-*funded research project (possibly jointly with 4 above)*
6	The current general lack of systematic data to rank existing, known allergenic proteins according to their allergenic potency reflects a significant knowledge gap, which impairs the development and validation of potential methodologies. This could be addressed by investigating responses to homologous series of proteins with different allergenicity, using as a starting point the ImpARAS work on protein pairs *How: European*-*funded research project*
7	No single distinct molecular parameter (or pattern) within one protein family seems to be exclusively responsible for the allergenic potential at the site of elicitation. However, continued detailed characterisation of allergens may further elucidate molecular pattern, which present intrinsic adjuvanticity, that further stimulate the immune system towards an increased efficiency in sensitisation against the allergenic protein *How: European*-*funded research project (possibly joint with 6 above)*
8	Better knowledge on the impact of different food matrices and food processing on allergenicity of dietary proteins. In addition, the impact of the interaction of food allergens with food components on allergenicity is not fully understood *How: European or national*-*funded Research project*

As illustrated in Table 4, European-funding plays a major role, because this topic is of public interest throughout Europe and food safety is regulated at the European level. Furthermore, the needs are pre-competitive rather than commercial. It is founded on the premise that newly introduced food proteins must not increase the already high burden of allergies on society and an urgent need to expedite the introduction of sustainable, nutritious and safe food to the market.

To enable the safe introduction of novel and more sustainable food protein sources, while protecting humans from unacceptable food allergy risks, we need to better predict the potential allergenicity of novel proteins. The GMO EFSA panel [8], ETP Food for life [9] and COST Action ImpARAS stress that a transparent, evidence-based, validated, allergenicity risk assessment based on novel methodologies is a necessity and currently hampers the introduction of novel sustainable foods on the market. This was confirmed during the ImpARAS stakeholder meeting, held on 26^th^ of November 2018 in Brussels, by the representatives from DG Research & Innovation, Unit F3—Agri-Food Chain, ETP Food for life, EFSA, Europabio and FooddrinkEurope.

The ImpARAS COST Action aimed to arrive at a consensus between different stakeholders (e.g., industry, regulators, scientists) to progress in allergenicity risk assessment. We hope that this Action will be the beginning of new collaborations and research projects that will ultimately lead to an improved allergenicity risk assessment strategy for novel food proteins.

## Data Availability

Not applicable.

## Appendix

See Table 5.

**Table 5:** List of most relevant peer-reviewed papers from the ImpARAS network

No	Title	Reference (DOI)
1	Current (food) allergenic risk assessment: is it fit for novel foods? status quo and identification of gaps	Mazzucchelli (2018) (10.1002/mnfr.201700278)
2	Application of the Adverse Outcome Pathway (AOP) concept to structure the available in vivo and in vitro mechanistic data for allergic sensitisation to food proteins	Van Bilsen (2017) (10.1186/s13601-017-0152-0)
3	Current challenges facing the assessment of the allergenic capacity of food allergens in animal models.	Bøgh (2016) 10.1186/s13601-016-0110-2
4	Allergenicity risk assessment of new or modified dietary proteins: a critical review of current strategies.	Remington (2018) 10.1016/j.fct.2017.12.025
5	Experimental food allergy models to study the role of innate immune cells as initiators of allergen specific Th2 immune responses	Hussain (2015) 10.1016/j.ddmod.2016.08.001
6	The use of animal models to discover immunological mechanisms underpinning sensitization to food allergens	Smit (2015) 10.1016/j.ddmod.2016.09.001
7	A review of animal models used to evaluate potential allergenicity of genetically modified organisms (GMOs)	Marsteller (2015) 10.1016/j.ddmod.2016.11.001
8	In silico tools for exploring potential human allergy to proteins.	Hayes (2015) 10.1016/j.ddmod.2016.06.001
9	Non-IgE mediated food allergy	Lozano-Ojalvo (2015) 10.1016/j.ddmod.2016.09.003
10	Applicability of epithelial models in protein permeability/transport studies and food allergy	Cubells-Baeza (2015) 10.1016/j.ddmod.2016.08.002
11	Epithelial models to study food allergen induced barrier disruption and immune activation	Gavrovic-Jankulovic (2015) 10.1016/j.ddmod.2016.09.002
12	IgE—the main player of food allergy	Broekman (2015) 10.1016/j.ddmod.2016.07.001
13	Influence of microbiome and diet on immune responses in food allergy models	Barcik (2015) 10.1016/j.ddmod.2016.06.003
14	Static and dynamic in vitro digestion models to study proteins stability in the gastrointestinal tract	Dupont (2015) 10.1016/j.ddmod.2016.06.002
15	Kiwifruit cysteine protease actinidin compromises the intestinal barrier by disrupting tight junctions	Grozdanovic (2016) 10.1016/j.bbagen.2015.12.005
16	Glycation of the major milk allergen β‐lactoglobulin changes its allergenicity by alterations in cellular uptake and degradation	Perusko (2018) 10.1002/mnfr.201800341
17	Proteomics in food: quality, safety, microbes and allergens.	Piras (2016) 10.1002/pmic.201500369
18	Allergenic and novel food proteins: state of the art and challenges in the allergenicity assessment	Pali-Schöl (2018) 10.1016/j.tifs.2018.03.007
19	Cross-reactivity in fish allergy: a double-blind, placebo-controlled food-challenge trial	Sørensen (2017) 10.1016/j.jaci.2017.03.043
20	Important plant food allergens (part I): what is shaping their allergenic potency—physicochemical properties and beyond	Costa, In Prep
21	Important animal food allergens (part II): what is shaping their allergenic potency—physicochemical properties and beyond	Costa, In prep
22	The relevance of a digestibility evaluation in the allergenicity risk assessment of novel proteins. Opinion of a joined initiative of COST Action ImpARAS and COST Action INFOGEST	Verhoeckx (2019) 10.1016/j.fct.2019.04.052
23	Applying the adverse outcome pathway (AOP) for food sensitization to support in vitro testing strategies	Lozano-Ojalvo (2019) 10.1016/j.tifs.2019.01.014
24	Overview of in vivo and ex vivo endpoints in murine food allergy models: suitable for evaluation of the sensitizing capacity of novel proteins?	Castan (2020) 10.1111/all.13943
25	Defining the targets for the assessment of IgE-mediated allergenicity of new or modified food proteins	Houben (2019) 10.1016/j.fct.2019.02.036
26	Jug r 6 is the allergenic vicilin present in walnut responsible for IgE cross-reactivities to other tree nuts and seeds	Dubiela (2018) 10.1038/s41598-018-29656-4
27	Fish-allergic patients tolerate ray based on the low allergenicity of its parvalbumin	Kalic (2018) 10.1016/j.jaip.2018.11.011
28	Homologous tropomyosins from vertebrate and invertebrate: recombinant calibrator proteins in functional biological assays for allergenicity assessment of novel animal foods	Klueber (2020) 10.1111/cea.13503
29	Hypothesis paper/concept introduction: is it possible to establish a generic threshold of exposure for allergic sensitization to food proteins	Bernard Madsen, In prep

## Abbreviations

AOP: Adverse Outcome Pathway
BAT: Basophil Activation Test
COST: Cooperation in Science and Technology
DCs: Dendritic Cells
EC: European Commission
EFSA: European Food Safety Authority
GI: Gastrointestinal
ImpARAS: Improved Allergenicity Risk Assessment Strategy
GMO: Genetically Modified Organisms
IgE: Immunoglobulin of type e
ILC2: Innate Lymphoid cells type 2
KE: Key event
MIE: Molecular Initiation Event
nsLTPs: Non-specific Lipid Transfer Proteins
OECD: Organisation for Economic Co-operation and Development
PR10: Pathogenesis-Related protein family 10
SAAOP: Society for the Advancement of Adverse Outcome Pathways
STSM: Short-Term Scientific missions
TTC: Threshold of Toxicological Concern
